# Ileal Tuft Cell Depletion Is Associated With Preterm Necrotizing Enterocolitis

**DOI:** 10.1016/j.gastha.2025.100744

**Published:** 2025-07-11

**Authors:** Shirley Wang, Addison Franca, Adam Wilson, Hala Chaaban, Kathryn Y. Burge

**Affiliations:** Section of Neonatal-Perinatal Medicine, Department of Pediatrics, University of Oklahoma Health Sciences Center, Oklahoma City, Oklahoma

Necrotizing enterocolitis (NEC) is a devastating inflammatory disorder of the preterm infant intestine, associated with an incidence of 7%–10% in very low birthweight infants (<1,500 g) and a mortality approaching 25%–30%.^1^ While NEC pathogenesis is poorly understood, a combination of risk factors, including prematurity, formula feeding, and dysbiosis,^2^ induce distal small bowel inflammation, necrotic skip lesions, systemic inflammation, multiorgan failure, and death. Compared with the intestine of term infants, that of preterm infants at risk of NEC is characterized by heightened permeability, elevated expression of inflammatory mediators, and fewer mucus-producing goblet cells.^3^ These features, in addition to microvascular irregularities and episodic hypoxia, contribute to mucosal injury in the preterm NEC intestine.

Intestinal tuft cells (TCs), specialized, low-abundance, epithelial chemosensory cells, are an enigmatic and heterogeneous cell type. With an emerging role as mediators of host-microbial crosstalk, TCs are best known for inducing a type 2 immune response to helminth or protist infection, reducing host epithelial contact with luminal pathogens through a circuit involving innate lymphoid cells type 2 (ILC2s), collectively termed the “weep and sweep” response.^4^ Small intestinal TCs express a panoply of apical receptors with which to monitor and integrate microbial metabolite signals. Ligand-binding of these intestinal TC receptors activates an intricate signal transduction pathway, facilitating epithelial release of immunomodulatory effectors. In mice, the canonical intestinal TC effector, anti-inflammatory interleukin (IL)-25, induces the TC-ILC2 circuit, with ILC2 release of IL-13 directing, among many other effects, expansion of TCs and goblet cells^5^ within the intestinal epithelium.

Due to the inherent complexity of the TC-ILC2 circuit, our current understanding of intestinal TC biology is predicated nearly entirely on adult mouse models, with major effectors within the human intestinal TC-ILC2 circuit yet to be identified. In piglets, a model for human intestinal development, TC numbers peak at birth at 25% of the epithelium.^6^ Early TC emergence in physiologically similar mammals, combined with the knowledge that human ILC2s infiltrate the fetal intestine and peak in concentration during early infancy,^7^ indicates the potential importance of the TC-ILC circuit to the fetal-neonatal transition. However, while intestinal TCs arise during the second trimester,^8^ TC relative abundance within the human epithelium during infancy is not known.

Importantly, pathogenesis of adult Crohn’s disease, an inflammatory condition sharing many NEC features, is characterized by a significant loss of ileal TCs.^9^ In mouse models, small intestinal TCs are most prevalent and inducible in the distal ileum,^9^ the most common site of NEC injury. However, despite known functions in the adult as major surveyors of gut microbial signals and the fact many G protein-coupled receptors are druggable targets, TCs, have not been evaluated in the context of NEC pathogenesis. Using immunofluorescent microscopy, this study aimed to determine the extent to which TCs are present during gastrointestinal development in infancy, and the degree to which ileal TC numbers may be altered in association with NEC pathogenesis.

Deidentified tissues from small intestinal surgical resections of patients with NEC or controls (eg, intestinal atresia; Table A1) were immunofluorescently stained. TCs were identified by costaining of the established adult human TC markers,^10^ epidermal growth factor receptor phosphotyrosine 1068 (p-EGFR) and β-actin (Figure 1D, Figure A1C), as well as by the characteristic flask-shaped cell morphology. Immunofluorescent advillin staining (Figure 1E) was also performed to confirm these results. Our data indicate ileal TCs are at least twice as abundant in the healthy infant epithelium as has been reported in the adult (4.5% vs <1%, *P* < .0001; Figure 1A and B),^11^ potentially not surprising as the TC signaling partner, ILC2s, are enriched within the neonatal intestine.^7^ We then compared control TC numbers with those of NEC tissues, demonstrating preterm infant TCs are obliterated in the NEC ileum at the time of surgical resection (Figure 1A and B, Figure A1). Further, the apical region of control TCs was characterized by significantly greater p-EGFR staining intensity compared to NEC TCs (*P* = .0051; Figure A1B), the latter of which appeared to have more diffuse p-EGFR staining within the cytoplasm. TCs in both tissues were often visualized in clusters of 4–5 cells, likely indicating expansion via autocrine, rather than paracrine, means. Finally, in healthy control tissue, TCs were significantly more common within the small intestinal villus rather than the crypt (Figure 1C), suggesting a potential prioritization toward chemosensing of luminal metabolites over potential tissue regeneration^8^ in neonates.

**Figure:**
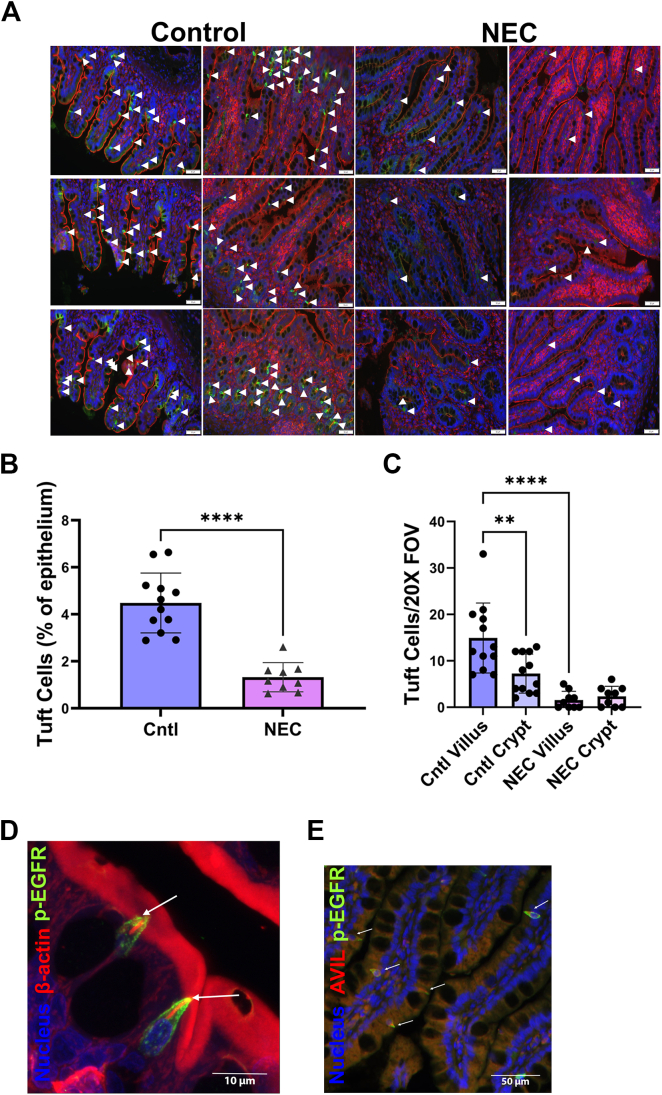
TCs are depleted within the distal small intestine of neonates with NEC. (A) Representative control and NEC TC staining (see *Methods*), demonstrating striking depletion of TCs in NEC patient tissues (p-EGFR—AF488; β-actin—AF647; nuclei—Hoechst 33342; scale bar = 50 μm); (B) Difference in TCs as a percentage of total epithelial cells in control and NEC distal small intestine (unpaired t-test with Welch’s correction). Each point represents the percentage of TCs within the epithelium in a single patient 20X FOV; *n* = 4 control, *n* = 3 NEC, 3 20X FOVs/patient; (C) Distribution of TCs in crypts and villi of control and NEC tissues (ANOVA with Tukey’s post-hoc). Each point represents the TC count in a single patient 20X FOV; *n* = 4 control and *n* = 3 NEC; 3 20X FOVs/patient; (D) Representative β-actin microfilament ‘rope’ (arrows) at the apical end of control TCs (p-EGFR—AF488; β-actin—AF647; nuclei—Hoechst 33,342); (E) Representative costaining of AVIL and p-EGFR within the neonatal ileum (p-EGFR—AF488; AVIL—AF647; nuclei—Hoechst 33342; magnification x40 obj.; scale bar = 50 μm). (B, C) ∗∗*P* < .01, ∗∗∗∗*P* < .0001. TC, tuft cell; NEC, necrotizing enterocolitis; p-EGFR, epidermal growth factor receptor phosphotyrosine 1068; AVIL, advillin; FOV, field of view.

This study is subject to several significant limitations. Because NEC is a rare disease, our sample size is modest, but still provides significant power with which to discriminate differences in NEC and control tissues. While we age-matched controls to the greatest extent possible, the most common surgical bowel indication in preterm infants is NEC, and thus the gestational age of our control infants was significantly older. Because these were historical biobanked samples, prospective collections in the future will evaluate potential associations between TC numbers and microbiome signatures. Finally, this study implicates an association, rather than causation. Future studies will attempt to determine whether TC loss is a pathophysiological feature versus a consequence of NEC.

In conclusion, we have demonstrated TCs are abundantly present in the noninflamed infant intestine, and that NEC pathogenesis in preterm infants is associated with loss of ileal TCs, together pointing to a potential role for the oft-neglected intestinal TC in establishment of mucosal immunity during neonatal development. Given the recent findings that expansion of murine TCs resolves ileal inflammation,^9^ and that human TCs may serve as a regenerative population within the epithelium postinjury,^8^ modulation of the intestinal TC-ILC2 circuit may provide novel avenues for prevention and treatment of NEC in preterm infants.

## Figures and Tables

**Supplementary Figure S1:**
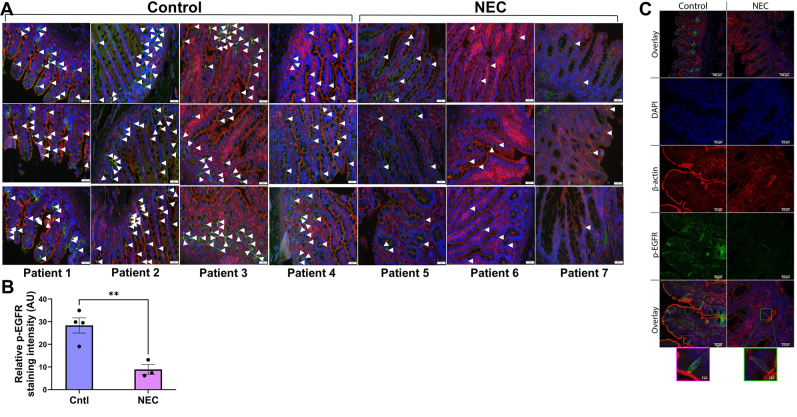
(A) Control and NEC tuft cell staining for all 7 patients, demonstrating striking depletion of tuft cells in NEC patient tissues (p-EGFR [green], β-actin [red], nuclear [blue]; scale bar = 50 μm); (B) Difference in apical p-EGFR staining in control and NEC tissues (unpaired t-test with Welch’s correction; *n* = 4 control and *n* = 3 NEC; ∗∗ *P* < .01). Each point represents an average of 3 20X FOV/patient; (C) Representative human preterm infant control (left) and NEC (right) ileum, stained for p-EGFR Y1068 (green), β-actin (red), and nucleus (blue). Magnification (x20 obj., scale bar = 100 μm) in top row, magnification (x63 obj., scale bar = 25 μm) in second thru fifth rows and magnification (x315 obj., scale bar = 5 μm) in bottom insets. NEC, necrotizing enterocolitis; p-EGFR, epidermal growth factor receptor phosphotyrosine 1068.
