# Supplementary material for: Ileal Tuft Cell Depletion Is Associated With Preterm Necrotizing Enterocolitis
Source: Gastro Hep Adv. 2025 Jul 11;4(10):100744. doi: 10.1016/j.gastha.2025.100744 (PMC12444462; doi:10.1016/j.gastha.2025.100744)
Supplement: Supplementary table S1 [file mmc1.pdf]

	Patient 1	Patient 2	Patient 3	Patient 4	Patient 5	Patient 6	Patient 7	*P*
**Intestinal Disease**								
Diagnosis	Distal Small Bowel Atresia	Distal Small Bowel Atresia	Ileal Atresia	Jejunal Atresia	NEC	NEC	NEC	N/A
Bell's Stage	N/A	N/A	N/A	N/A	IIIB	IIIB	IIIA	N/A
Pathology Report	Focal luminal obliteration by fibrosis	Small intestinal atresia with one blind end	Focal mucosal congestion/hemorrhage/bowel adhesions	One blind end, mild serosal fibrosis	Subacute NEC with focal perforation and marked acute/chronic inflammation	Multifocal transmural necrosis and acute and chronic inflammation	Transmural acute and chronic inflammation with focal perforation	N/A
**Demographics**								
Sex	Male	Male	Male	Female	Male	Female	Male	>.9999
Race	W	W	H, Latino	W	W	H, Latino	W	.0955
GA (Delivery), wk	35 5/7	39 1/7	40 1/7	35 2/7	26 4/7	27 2/7	27 2/7	**.0008**
Birthweight, g	2910	2980	3740	2370	870	1164	990	**.0021**
GA (Surgery), wk	42 1/7	39 3/7	40 3/7	39 3/7	30 3/7	30 5/7	31 5/7	**<.0001**
**Perinatal Metrics**								
Maternal Drug Use	No	Yes (Narcotics/Tobacco)	No	No	No	No	No	>.9999
Mode of Delivery	Vaginal	C-Section	Vaginal	C-Section	Vaginal	C-Section	C-Section	>.9999
Apgar Score at 5 min	9	9	9	9	7	7	9	.0624
**Comorbidities**								
Other Comorbidities	SVT	N/A	N/A	N/A	N/A	PDA, small pulmonary hemorrhage	1p36 Deletion Syndrome	N/A
**Interventions**								
Transfusions <48 h before Diagnosis	No	No	No	No	Unknown	No	No	>.9999
Respiratory Support, Pre-Diagnosis	RA	RA	RA	RA	CMV	NC	NC	N/A
Respiratory Support, Post-Diagnosis	RA	RA	RA	CMV	HFV	CMV	CMV	N/A
**NICU Metrics**								
Feed Type/Volume	NPO	NPO	NPO	NPO	Unknown	DHM with bovine fortifier/FF	Unknown	N/A
Hb/Hct, g/dL/%	16.4/ 48	14.5/ 41.7	13.2/37.6	13.1/ 39.2	14.5/ 43.1	10/30	13.4/38	.3034/ .3223
WBC ($x10^3/mm^3$)	13.2	10.3	12.5	21	2.18	3.8	14.4	.1383
Platelet Nadir w/in 48 h of Diagnosis, per µL	231,000	330,000	273,000	329,000	384,000	149,000	39,000	.3154
Lymphocytes, %	20	13	30	45	52	35	44	.1292
Neutrophils, %	69	74	64	29	30	20	26	**.0411**
Monocytes, %	2	13	3	10	9	26	15	.1240
**Surgical Metrics**								
Sample Location	Jejunoileum	Jejunoileum	Ileum	Distal Jejunum	Ileum	Ileum	Ileocecum	N/A
Length Resected, cm	8	17	28	11.5	11.4 ileum, 2.2 colon/ICV	Most (<30 cm remaining from small bowel and colon)	1.5 ileum, 3 colon	N/A
**Clinical Outcome**								

Survival (cause of death)	Yes	Yes	Yes	No (respiratory failure at 1 yr)	Yes	Yes	Yes	>.9999
GI Outcomes	Discharged on FF		Ileostomy; Discharged on FF		G-Tube; Ostomy	SGS; G-Tube	Discharged on FF	N/A

Table S1. Patient clinical and demographic characteristics. Significance (bolded, $p < 0.05$) was determined by unpaired, two-tailed T-tests or Fisher's exact tests, as appropriate. Abbreviations: NEC: necrotizing enterocolitis; W: White; H: Hispanic; GA: gestational age; C-Section: Cesarean section; SVT: superventricular tachycardia; PDA: patent ductus arteriosus; RA: room air; CMV: conventional mechanical ventilation; NC: nasal cannula; HFV: high-frequency ventilation; NPO: nil per os; DHM: donor human milk; FF: full feeds; Hb: hemoglobin; Hct: hematocrit; WBC: white blood cells; ICV: ileocecal valve; G-tube: gastrostomy tube; SGS: short gut syndrome.
