# Supplementary material for: Ileal Tuft Cell Depletion Is Associated With Preterm Necrotizing Enterocolitis
Source: Gastro Hep Adv. 2025 Jul 11;4(10):100744. doi: 10.1016/j.gastha.2025.100744 (PMC12444462; doi:10.1016/j.gastha.2025.100744)
Supplement: Supplemental Material [file mmc2.docx]

**Materials and Methods**

***Human preterm infant samples.*** Formalin-fixed, paraffin-embedded (FFPE) blocks of preterm infant samples from surgically resected ileum or jejunum from infants with necrotizing enterocolitis (NEC, *n* = 3) or controls (*e.g.*, intestinal atresia; *n* = 4) were obtained from the University of Oklahoma Health Sciences Center (OUHSC) Department of Pathology biobank (Institutional Review Board [IRB] protocol #16114), along with deidentified patient data and pathology reports. Samples were taken from the ‘healthy’ margins of resected NEC or atretic tissue. In some cases (*e.g.*, control patients 1 and 2), the exact location of the small bowel atresia was not specified, though histology dictates distal (*i.e.*, jejunum or ileum) small bowel.

***Immunofluorescence (IF)***

FFPE tissues were sectioned (5 µm) before deparaffinization and antigen retrieval. Slides were deparaffinized in xylene and rehydrated in ethanol. Antigen retrieval was performed by boiling slides in citrate buffer (10 mM, pH 6.0, Tween 0.05%) in a water bath for 20 min. Slides were cooled to room temperature (RT) and rinsed in phosphate buffered saline (PBS) Tween 0.05%. To preserve antibodies, tissue sections on slides were circled with an ImmEdge Hydrophobic Barrier PAP Pen (H-4000, Vector Laboratories). Slides were blocked and permeabilized overnight at 4°C in permeabilization/blocking buffer (2.5% bovine serum albumin [BSA], 2.5% donkey serum, 0.5% Triton X-100). The primary antibodies, Alexa Fluor^®^ 488 anti-EGFR phospho Y1068 (ab205827, Abcam) and CoraLite^®^ Plus 647-conjugated β-actin (CL647-66009, ProteinTech), were diluted 1:100 in permeabilization/blocking buffer and incubated overnight at 4°C. The slides were washed three times in PBS Tween 0.05%.

After washing, autofluorescence was quenched in quenching buffer (0.3 M glycine in deionized water, 5 mM CuSO_4_ in 50 mM amino acetate) for 90 min at RT. After two rinses with PBS, the slides were stained for nuclei with Hoechst 33342 (62249, ThermoFisher) for 5 min at RT. After washing twice with PBS, fluorescence images were acquired using a confocal microscope (Leica SP8). Images were formatted using Leica LAS X Office v.1.4.5.27713.

***Immunohistochemistry (IHC)***

Five μm tissue sections were deparaffinized in xylene and rehydrated in ethanol. Antigen retrieval was performed by heating the slides in a microwave oven for 10 min at power level 2 in 10 mM Tris base, 1 mM ethylenediaminetetraacetic acid (EDTA), 0.05% Tween-20 (pH 9.0) buffer. Slides were cooled to RT and rinsed in PBS Tween 0.05%. To preserve antibodies, tissue sections on slides were circled with an ImmEdge Hydrophobic Barrier PAP Pen and endogenous peroxidases were blocked by incubation with 3% hydrogen in PBS for 15 min at RT. Nonspecific proteins were blocked with a background sniper (B5966H, Biocare Medical) for 5 min at RT. The primary antibody anti-EGFR phosphor Y1068 (ab40815, Abcam) was diluted 1:100 in PBS and incubated overnight at 4°C. Subsequently, the slides were washed three times (10 min each) in PBS Tween 0.05%. Goat anti-rabbit IgG conjugated to horseradish peroxidase (HRP) secondary antibody (31460, ThermoFisher) was diluted 1:200 in PBS and incubated for 1 h at RT. The slides were then washed three times (10 min each) in PBS Tween 0.05%. 3,3-Diaminobenzidine (DAB) (34002, ThermoFisher) was used as the chromogen (brown). The slides were counterstained with hematoxylin, dehydrated in ethanol, cleared in xylene, and mounted for observation. For cell counts, p-EGFR Y1068^+^ cells and total epithelial cells were counted from randomly selected 20X fields of view (FOV) using the ImageJ Plugins > Analyze > Cell Counter. Results were expressed as the percentage of p-EGFR Y1068^+^ cells per total epithelial cells. Phosphorylated EGFR staining intensity was quantified in ImageJ. The freehand tool was used to select a region of interest incorporating the apical region of TCs. Analyze > Measure was used to obtain the mean value, from which the background intensity was subtracted. Intensity values were then averaged across multiple fields of view/patient.

***Statistics.*** Statistical analysis for clinical and demographic information was conducted using GraphPad Prism v.10.4.1, using unpaired two-tailed t-tests and Fisher’s exact tests, as appropriate. Statistics for IF and IHC were performed using ImageJ with unpaired, two-tailed Student's t test and Welch’s correction. Error bars represent mean ± SD (standard deviation). *P*-values ​​less than .05 were considered statistically significant.

**Figure A1.** **(A)** (A) Control and NEC tuft cell staining for all 7 patients, demonstrating striking depletion of tuft cells in NEC patient tissues (p-EGFR [green], β-actin [red], nuclear [blue]; scale bar = 50 µm); (B) Difference in apical p-EGFR staining in control and NEC tissues (unpaired t-test with Welch’s correction; n = 4 control and n = 3 NEC; ** P < .01). Each point represents an average of 3 20X FOV/patient; (C) Representative human preterm infant control (left) and NEC (right) ileum, stained for p-EGFR Y1068 (green), β-actin (red), and nucleus (blue). Magnification (x20 obj., scale bar = 100 µm) in top row, magnification (x63 obj., scale bar = 25 µm) in second thru fifth rows and magnification (x315 obj., scale bar = 5 µm) in bottom insets. NEC:, necrotizing enterocolitis; p-EGFR:, epidermal growth factor receptor phosphotyrosine 1068.
